# Time Series Data Augmentation for Energy Consumption Data Based on Improved TimeGAN

**DOI:** 10.3390/s25020493

**Published:** 2025-01-16

**Authors:** Peihao Tang, Zhen Li, Xuanlin Wang, Xueping Liu, Peng Mou

**Affiliations:** 1 Division of Advanced Manufacturing, Tsinghua Shenzhen International Graduate School, Tsinghua University, Shenzhen 518055, China; tph22@mails.tsinghua.edu.cn (P.T.); zhen-li22@mails.tsinghua.edu.cn (Z.L.); wang-xl23@mails.tsinghua.edu.cn (X.W.); 2 Department of Mechanical Engineering, Tsinghua University, Beijing 100084, China; moupeng@tsinghua.edu.cn

**Keywords:** data augmentation, TimeGAN, deep learning, time series

## Abstract

Predicting the time series energy consumption data of manufacturing processes can optimize energy management efficiency and reduce maintenance costs for enterprises. Using deep learning algorithms to establish prediction models for sensor data is an effective approach; however, the performance of these models is significantly influenced by the quantity and quality of the training data. In real production environments, the amount of time series data that can be collected during the manufacturing process is limited, which can lead to a decline in model performance. In this paper, we use an improved TimeGAN model for the augmentation of energy consumption data, which incorporates a multi-head self-attention mechanism layer into the recovery model to enhance prediction accuracy. A hybrid CNN-GRU model is used to predict the energy consumption data from the operational processes of manufacturing equipment. After data augmentation, the prediction model exhibits significant reductions in RMSE and MAE along with an increase in the R^2^ value. The prediction accuracy of the model is maximized when the amount of generated synthetic data is approximately twice that of the original data.

## 1. Introduction

As environmental issues such as global warming become increasingly severe, controlling large energy consumption sources like manufacturing processes has emerged as a prominent area of research. Predicting the energy consumption time series of manufacturing equipment enables manufacturers to gain a more accurate understanding of energy consumption during the manufacturing process, allowing for the formulation of more rational energy management strategies. Additionally, it assists enterprises in identifying abnormal fluctuations in energy consumption in advance, thereby ensuring the stability of the production process [1]. Methods for predicting time series can be categorized into statistical approaches, classical machine learning methods, and deep learning methods [2]. Among these, deep learning methods train the multi-layer network structure through existing data and mine the multidimensional features of the data to express abstract semantic information, thus realizing the accurate prediction of time series [3].

However, the performance of deep learning algorithms is significantly influenced by the quality of the training dataset. In practical manufacturing processes, obtaining a substantial amount of authentic and effective time series data is challenging due to issues such as missing historical data, instability during data collection, and noise interference [4]. When the training data are insufficient, the accuracy of the prediction model may fail to meet practical requirements. Data augmentation is an effective approach to address the issue of inadequate training data in small sample learning. By learning and reorganizing the characteristics of existing data, it can generate samples with similar characteristics to the original samples, thus enhancing the generalization ability and robustness of the prediction model. Based on the principles of time series augmentation methods [5], they can be categorized into rule-based approaches, machine learning methods, and deep learning methods.

Rule-based methods for data generation utilize fixed conditions to constrain the attributes and structural characteristics of the generated time series data. Common constraints include parameters that reflect statistical features of the data, such as value ranges, trends, and degrees of dispersion. These methods do not need a lot of historical data to train the generation model, and the generated results are well interpretable. However, the characteristics of the generated data are relatively simple, and it is impossible to accurately simulate the complex data change trend. Kang et al. [6] proposed a time series generator based on a mixed auto-regressive model, constructing the data generation model through a mixture of Gaussian AR models and employing a genetic algorithm to adjust the parameters of the MAR model, effectively generating a controllable time series. Zhou et al. [7] expanded the original dataset by introducing noise and randomly setting partial data to be missing. Additionally, models such as frequency domain transformation [8] and Monte Carlo methods [9] have also been utilized for time series generation.

Traditional machine learning methods train models using historical time series and subsequently utilize the trained models to generate fitted time series. Common machine learning methods employed for time series generation include auto-regressive models [10], clustering models [11], and Bayesian networks [12]. Shamshad et al. [13] used first- and second-order Markov chain models to obtain a fitted time series according to the existing real-time sequence data and the unified random number generator. Li et al. [14] proposed a method of generating a time series with long-term correlation based on a Gaussian mixture model-hidden Markov model (GMM-HMM), which defined data correlation through discrete state variables within the hidden Markov model and established a mapping relationship with the Gaussian mixture model.

**Figure 1:**
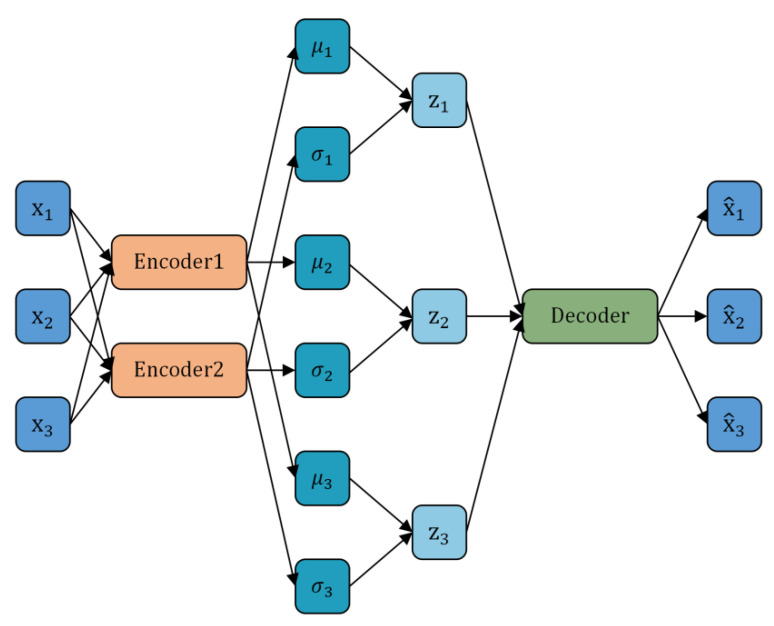
The structure of the Variational Autoencoder.

Compared to traditional machine learning models, deep learning models can capture more complex feature relationships from historical data, thereby generating more realistic time series. Among these, the Variational Autoencoder (VAE) and Generative Adversarial Network (GAN) are two mainstream models for time series generation. The Variational Autoencoder model [15] consists of two components: an encoder and a decoder, as shown in Figure 1. The encoder converts the original data into intermediate variables to extract the abstract features of the data, while the decoder transforms the intermediate variables back into the format of the original data. Chung et al. [16] developed a Variational Recurrent Neural Network (VRNN) model for time series generation by combining VAE with RNN. The Stochastic Recurrent Neural Network (SRNN) [17] builds upon the VRNN by integrating RNN and State Space Model (SSM) methods, separating deterministic and stochastic processes to enhance the fitting performance of the generated data. TimeVAE [18] extends the VAE framework by incorporating interpretable variables related to the temporal dimension in the data generation process and effectively reduces training time. However, VAE-based data generation methods have high requirements for prior knowledge and may struggle to accurately learn the distribution characteristics of complex data.

**Figure 2:**
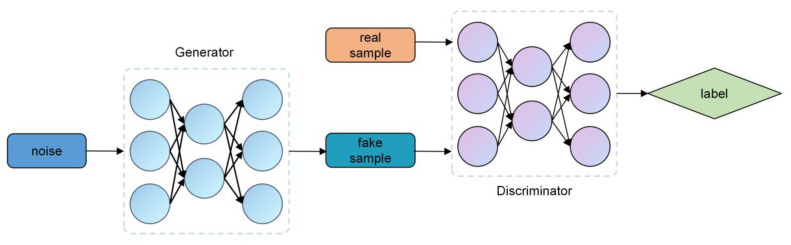
The structure of the Generative Adversarial Network.

The Generative Adversarial Network (GAN) is a deep learning model proposed by Goodfellow et al. [19] in 2014. The structure primarily consists of two components: the generator and the discriminator, as shown in Figure 2. The generator captures the distribution characteristics of the original data and generates new data, while the discriminator compares the generated data samples with the original data samples to determine the feature distance between the two. This feature distance is then used to update the parameters of the generator. This process is repeated until the discriminator finds it challenging to distinguish between the features of the generated data and the real data. Hadley et al. [20] utilized an improved Conditional Generative Adversarial Network (CGAN) to generate a kinematic time series, enhancing the model’s accuracy. Ramponi et al. [21] employed a Time Conditional Generative Adversarial Network (TCGAN) for time series data augmentation, modifying the conditional input to sampling timestamps, allowing for better fitting of irregularly spaced sampled data. RGAN and RCGAN [22] use RNN models as components for both the generator and the discriminator, enabling the generation of a realistic multidimensional time series. TimeGAN [23] introduces a supervised learning mechanism through a learnable embedding space, allowing the model to learn the features of the training data. COT-GAN [24] designs the objective loss function based on causal optimal transport and addresses the convergence issue of small-batch training datasets using an improved Sinkhorn differentiation operation. Additionally, variants of GANs, such as SeqGAN [25], DoppelGANger [26], RTSGAN [27], and DCCGAN [28], have also been employed for time series data generation.

Using data augmentation models to expand the training set of a time series can effectively enhance the model’s performance. However, research on the application of such methods to energy consumption data in manufacturing processes remains limited, and the potential of GAN models has yet to be fully explored. Given these issues, we propose an energy consumption time series augmentation algorithm based on an improved TimeGAN model. This approach enhances the model’s feature learning capability by incorporating an attention mechanism layer into the structure of the recovery model, and the generated data is used to expand the training dataset for deep learning prediction models. The contributions of this study are as follows: (1) the application of the TimeGAN model to address the issue of data scarcity in energy consumption during manufacturing processes; (2) improvements to the TimeGAN model structure to ensure that the statistical features of the generated data closely resemble those of the original data; and (3) the training of prediction models using the generated dataset, which effectively enhances the model’s prediction accuracy and explores the impact of generated data size on model performance.

This paper is organized as follows. Section 2 introduces the data augmentation model and prediction model used in this paper. Section 3 takes the equipment energy consumption data of the enterprise manufacturing process as an example to verify the characteristic difference between the generated data and the original data and the influence on the prediction model’s performance. Section 4 is the conclusion.

## 2. Methodology

### 2.1. Overview of the Method

**Figure 3:**
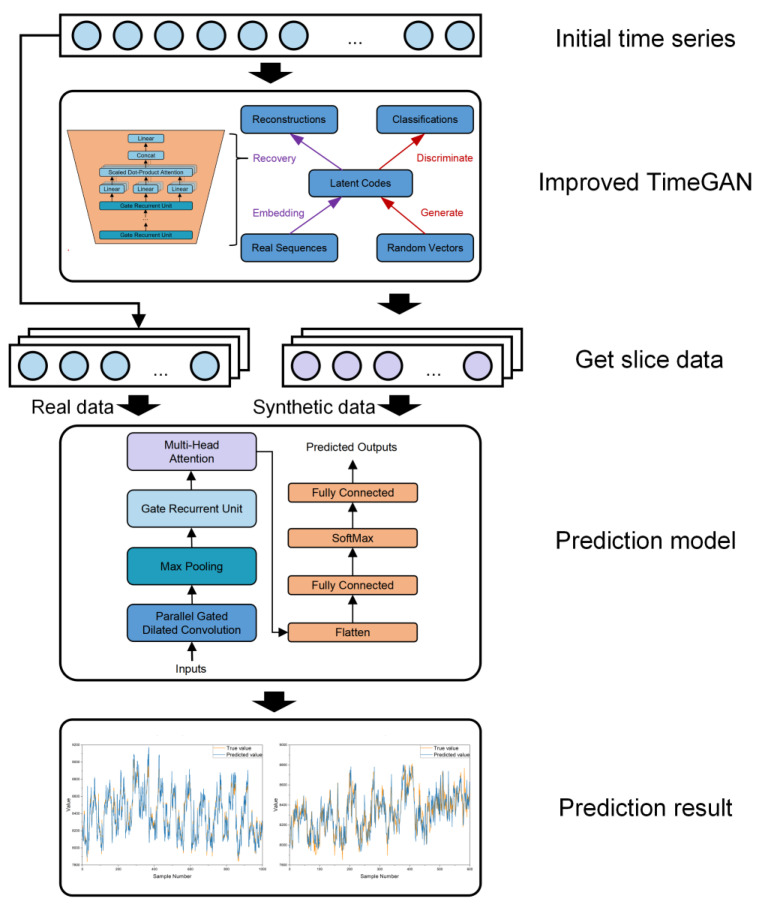
The research outline.

The training process and result verification process of the data augmentation model proposed in this paper are shown in Figure 3. Initially, the improved TimeGAN model is trained by using the existing real energy consumption data, and then the statistical characteristics of the generated data compared with the real data are calculated. Subsequently, the generated data are used to augment the training dataset of the CNN-GRU prediction model. The performance of this model is then compared with that of the prediction model trained on the original real dataset.

### 2.2. Improved TimeGAN Model

**Figure 4:**
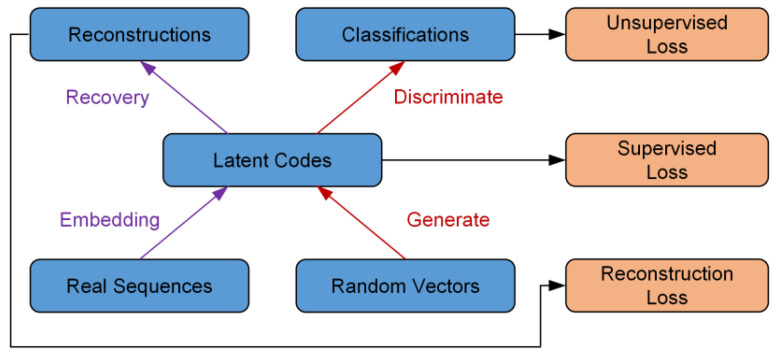
The structure of the TimeGAN model.

TimeGAN [23] is a variant of GAN proposed by Yoon et al. in 2019, demonstrating strong performance in generating time series. The network structure of the TimeGAN model is shown in Figure 4. The training model consists of four modules, comprising supervised embedding-recovery network training and unsupervised generate-discriminate network training. These two components adjust specific network parameters through three loss functions.

The embedding module and recovery module conduct supervised training of the model using actual sequence samples. During the encoding phase, the real sequences are transformed into latent codes through an embedding layer, which changes the data dimensionality while extracting the static and temporal features of the data. The shape of the input data is (N, T, F), where N is the number of samples, T is the time step, and F is the characteristic dimension. The embedding module changes its shape to (N, latent dim). The recovery module then reconstructs the data back to the original sequence dimensions, and the shape is (N, T, F). The generator and discriminator compete with each other through a zero-sum game mechanism. The generator fits the distribution characteristics of the original data to generate new data. At the same time, the discriminator compares the generated data samples with the original data samples to determine the feature distance between the two. This feature distance is then used to update the parameters of the generator. This process is repeated until the discriminator finds it hard to tell the difference between the features of the generated data and the actual data.

For a known distribution $Z\sim P_{Z}(z)$, such as a Gaussian or uniform distribution, we define a generator function G. The data generated by the generator are denoted as G(z), and the goal of the generator is to make the distribution of G(z) closely approximate the distribution of the real data $P_{Z}(z)$. To assess the similarity between these two distributions, a discriminator function D is defined and trained to determine whether the data are real. Therefore, the objective function for model training is defined as follows:

(1) $$\underset{G}{min}\underset{D}{max}L\left(D,G\right)=E_{x∼p(x)}\left[\ln D(x)\right]+E_{z∼p(z)}[ln(1-D(G(Z)))]$$

where E represents the mathematical expectation of actual data and random vectors, the generator function G can be any autoregressive model network, and the discriminator function D needs to realize the binary classification process.

**Figure 5:**
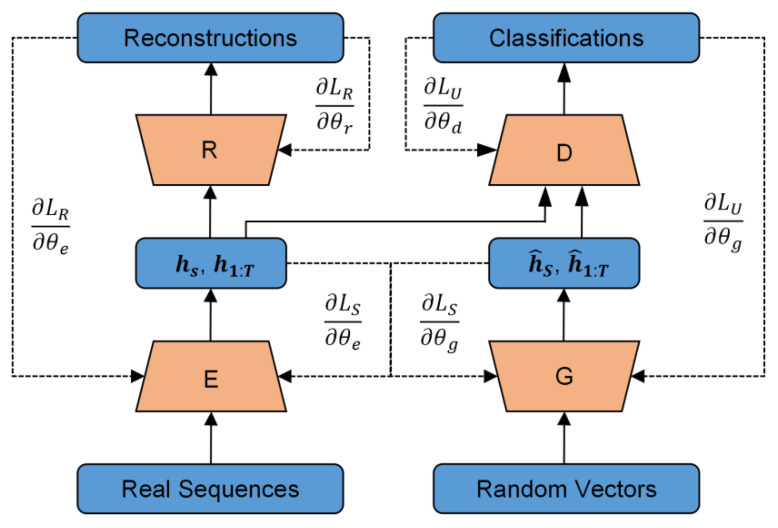
The loss functions of the TimeGAN model.

In the training process of the model, the four modules of the TimeGAN model iterate the training parameters through three loss functions, and the training process is shown in Figure 5. For the embedding-recovery structure, the characteristics of the data are extracted through the mapping relationship between the original data and the hidden space, and its loss function is defined as follows:

(2) $$L_{R}=E_{s,x_{1:T}\sim p}[{\left\|s-\tilde{s}\right\|}_{2}+\sum_{t}{\left\|x_{t}-{\tilde{x}}_{t}\right\|}_{2}]$$

For the generator module and discriminator module, it is necessary to make the generated result of the generator not be recognized as false data, and its loss function is defined as follows:

(3) $$L_{U}=E_{s,x_{1:T}\sim p}\left[logy_{s}+\sum_{t}logy_{t}\right]+E_{s,x_{1:T}\sim \hat{p}}\left[log(1-{\hat{y}}_{s})+\sum_{t}log(1-{\hat{y}}_{t})\right]$$

TimeGAN also includes a supervised training process. This is important because the characteristics of a time series are linked to its temporal attributes. Relying only on the embedding-recovery structure and the generator-discriminator structure may limit the model’s ability to recognize the temporal features of a time series, which may affect the quality of the generated data. During the training phase of the generator, the generator must produce the latent vector for the next time step based on the embedding of the actual time series $h_{1:t-1}$. Its loss function is defined as follows:

(4) $$L_{S}=E_{s,x_{1:T}\sim p}\left[\sum_{t}{\left\|h_{t}-g_{x}(h_{s},h_{t-1},z_{t})\right\|}_{2}\right]$$

**Figure 6:**
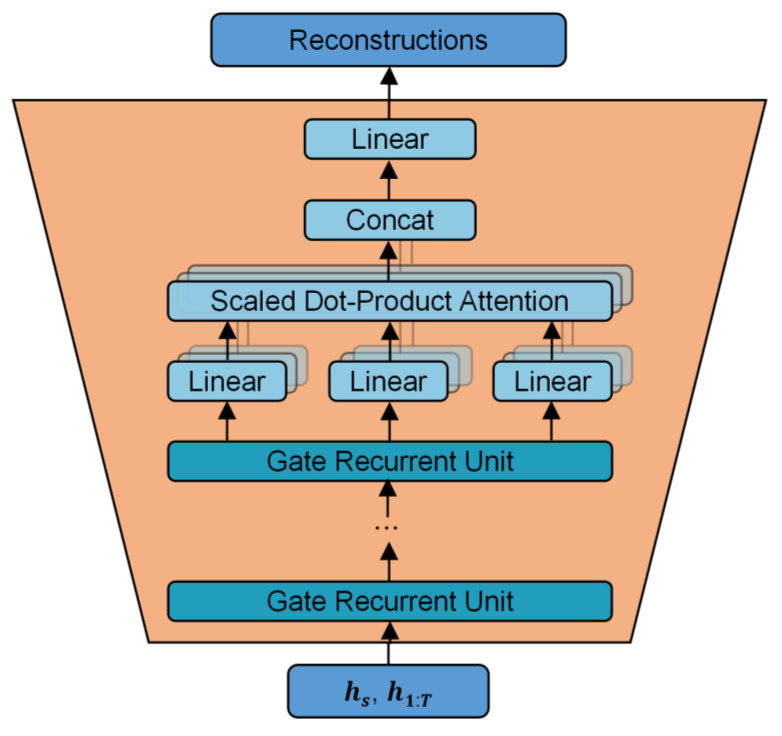
The structure of the improved recovery module.

The original TimeGAN model employs recurrent neural networks as the training model for the recovery module during time series augmentation. However, when the number of original data samples is small, this approach still struggles to learn dependencies effectively over longer time steps. Therefore, in the training process of the recovery module, an additional multi-head self-attention mechanism layer has been introduced after the existing structure, which utilizes multi-layer gated recurrent units for time series feature extraction. This enhancement aims to improve the model’s ability to learn key information from a time series and reduce the loss of information during training. The improved structure is shown in Figure 6.

The attention mechanism (AM) [28,29] was introduced by the Bengio team in 2014, and the self-attention mechanism proposed by the Google team in “Attention is All You Need” [29,30] has been widely utilized in the construction of deep learning models. The computation for the attention mechanism is defined as follows:

(5) $$head=Attention(Q,K,V)=softmax(\frac{QK^{T}}{\sqrt{d_{k}}})\cdot V$$

where Q, K, and V represent query, key, and value, respectively. After calculating the correlation between Q and K, the SoftMax function is applied for normalization to obtain the weight coefficients. Finally, the weighted value is computed to produce the final output result.

The multi-head self-attention mechanism is an improved version of the self-attention mechanism and is defined as follows:

(6) $$MultiHead(Q,K,V)=Concat({head}_{1},\ldots ,{head}_{h})W^{O}$$

It independently trains multiple self-attention mechanism models to calculate the self-attention heads and then combines the results of all of the attention heads and linearly transforms them to obtain the final output. This mechanism enables the model to pay more attention to the characteristic information of the data in different positions in a time series, which improves the model’s recognition performance and enhances the system’s robustness. The recovery module uses the RNN model for data analysis, but the effect of the recovery structure decreases with the increase in time step. The attention mechanism solves the problem of gradual information loss in the embedding-recovery process by giving different attention weights to the inputs in different time steps. By adding a multi-head self-attention mechanism layer to the recovery model, the model can extract the dependencies between time steps in the hidden state and give different weights to the final output time series, thus realizing the extraction of key information in the time series. The workflow of the modified recovery module is as follows: firstly, the data are transmitted to the multi-layer GRU model for time series feature extraction, and the output is calculated as Q, K, and V of the multi-head self-attention mechanism. Finally, the output sequence with the same length as the input sequence is generated by the output layer.

### 2.3. Prediction Model

**Figure 7:**
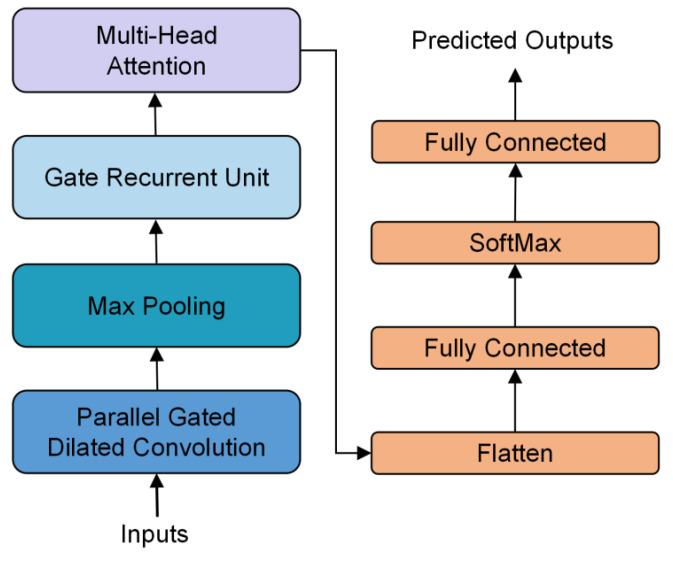
The structure of the prediction model.

The purpose of data augmentation is to improve the accuracy of the prediction model. This is achieved by expanding the training dataset used by the deep learning model when predicting the energy consumption time series in manufacturing processes. In the field of energy consumption data prediction, deep learning algorithms have good accuracy and feature-learning ability [31,32]. The structure of the deep learning model for energy consumption prediction is shown in Figure 7. First, the dataset is processed through a parallel gated dilated convolution layer for convolution. The input data are put into three parallel dilated convolution layers with different convolution kernel sizes to extract the feature information of different levels of the data. After the calculation of a layer of the gating unit, the outputs of the three are added together to obtain the final output of the CNN layer. Next, it goes through a max-pooling layer to reduce dimensionality and prevent model overfitting. The pooled feature data are input into a Gated Recurrent Unit (GRU) layer [33] to learn the temporal feature values. Then, the data are fed into a multi-head self-attention mechanism layer for weight allocation to extract important information. After the calculation result of the attention mechanism is transformed into one-dimensional data through the flattening layer, the final prediction result is obtained through two fully connected layers. A SoftMax layer is added between the two fully connected layers to help the model maintain numerical stability during training. The input time step length is set to 30, and the output time step length is set to 1. This indicates the prediction of the energy consumption value for the next time step.

## 3. Experiment

This paper validates the effectiveness of the designed data augmentation model using time series energy consumption data collected from sensors during the actual manufacturing process of a mobile phone manufacturing enterprise. In the data augmentation section, the comparative results of the statistical features between the data generated by the proposed model and the real data are presented. In the prediction model section, the variations in the prediction accuracy of the model are demonstrated under different amounts of generated data used to augment the training set, along with the selection of the optimal amount of generated data.

### 3.1. Data Source

**Figure 8:**
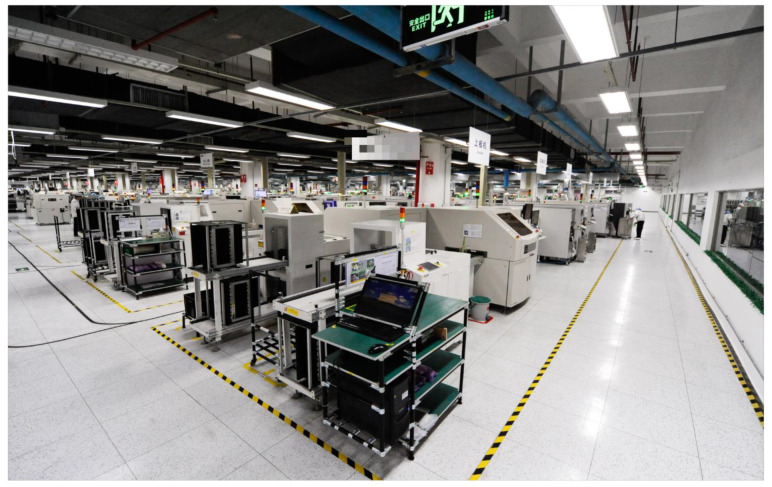
The manufacturing production line.

**Figure 9:**
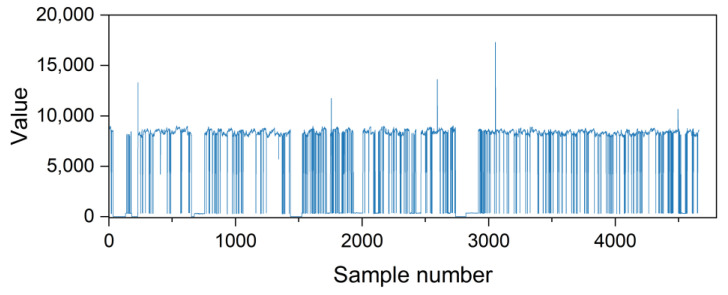
The energy consumption data of the board-splitting machine.

The dataset used in this paper came from a mobile phone production line of an electronic product manufacturing enterprise, with a schematic diagram of the production line shown in Figure 8. Smart meters installed along the production line collected the power consumption information of the equipment during operation, which was then converted into energy consumption values for analysis. Relevant energy consumption data for various devices on the production line were collected from 1 September 2024 to 22 October 2024. The energy consumption data from the board-splitting machine, which had higher energy consumption on the production line, were selected as the original dataset, and the fluctuations in the original data are shown in Figure 9. The energy consumption data were collected by the sensor every 15 min, and finally 4662 original data points were obtained, which were stored in a csv file for subsequent processing.

### 3.2. Data Preprocessing

**Figure 10:**
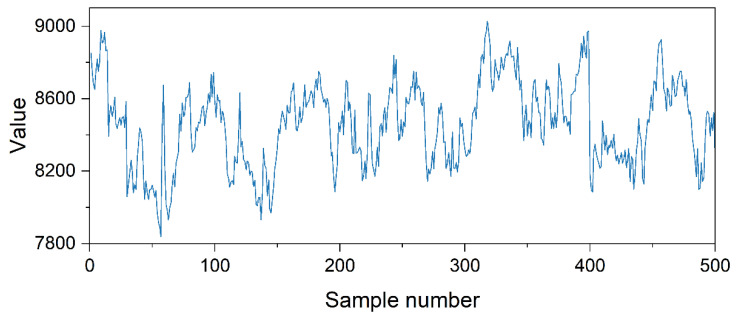
The time series after removing outliers.

Due to equipment failures and network transmission issues, the energy consumption data collected in practice often contain numerous outliers and erroneous values. Therefore, it is necessary to preprocess the original data to improve data quality before training the data augmentation model. The time series after removing identified outliers, based on the engineers’ experience, is shown in Figure 10. Min–max normalization was applied to scale the normal data to a range of 0 to 1, enhancing the model’s training performance while preserving the original data distribution. The normalized data were sampled using the sliding window method to generate the data fragments needed for training the model, and the sliding window size was set to 30 time steps.

### 3.3. Evaluation Metrics

For the data augmentation model, this paper uses various methods to evaluate the degree of fit between the generated data and the original data. To analyze the overall fluctuation trends of the data, the mean, standard deviation, quartiles, skewness, and kurtosis were calculated to determine whether both datasets exhibited the same statistical characteristics. They are defined as follows:

(7) $$Mean=\mu =\frac{1}{n}\sum_{i=1}^{n}x_{i}$$

(8) $$Standard\;deviation=\sigma =\sqrt{\frac{1}{n}\sum_{i=1}^{n}{{(x}_{i}-\mu )}^{2}}$$

(9) $$Skweness=\frac{n}{\left(n-1)(n-2\right)}\sum_{i=1}^{n}{\left(\frac{x_{i}-\mu }{\sigma }\right)}^{3}$$

(10) $$Kurtosis=\frac{n(n+1)}{\left(n-1)(n-2)(n-3\right)}\sum_{i=1}^{n}{\left(\frac{x_{i}-\mu }{\sigma }\right)}^{4}-\frac{3{\left(n-1\right)}^{2}}{\left(n-2)(n-3\right)}$$

The mean represents the central position of the data, reflecting the overall level of the dataset. The standard deviation measures the average distance of data points from the mean, indicating the degree of dispersion within the data. Quartiles divide the data into four parts, illustrating the distribution of the dataset. Skewness indicates the degree of asymmetry in the data distribution; a smaller skewness value suggests a more symmetric distribution. Kurtosis describes the sharpness of the data distribution; when kurtosis is greater than 3, it indicates the presence of a higher number of extreme values.

In order to better compare the distribution of original data and generated data, we used PCA and t-SNE to map a data fragment from 30-dimensional to 2-dimensional, so as to understand the distribution, clustering, and potential structure of data more intuitively. Principal Component Analysis (PCA) [34] is a widely used dimensionality reduction technique. It projects high-dimensional data into a lower dimensional space while preserving as much of the data’s variance as possible. The data are first standardized, and then the covariance matrix is computed. The covariance matrix undergoes eigenvalue decomposition to obtain the eigenvalues and their corresponding eigenvectors. The top k eigenvectors are selected based on the magnitude of the eigenvalues, which represent the principal components corresponding to the directions of maximum variance in the data. The original data are then projected onto the selected principal components to obtain the reduced dimensional data. PCA assumes that the data are linear and is effective in reducing dimensionality. T-Distributed Stochastic Neighbor Embedding (t-SNE) [35] is a nonlinear dimensionality reduction technique that minimizes the differences in similarity between data points in high-dimensional and low-dimensional spaces, thereby representing the characteristics of data points in a lower-dimensional space. For each pair of data points, $x_{i}$ and $x_{j}$, in a high-dimensional dataset, t-SNE first computes the conditional probabilities in the high-dimensional space to represent their similarity, as follows:

(11) $$p_{j|i}=\frac{exp({\left\|x_{i}-x_{j}\right\|}^{2}/2{\sigma_{i}}^{2})}{\sum_{k\neq i}exp({\left\|x_{i}-x_{k}\right\|}^{2}/2{\sigma_{i}}^{2})}$$

In processing low-dimensional data points, t-SNE uses a t-distribution to model the similarity between data points and computes their similarity as well, as follows:

(12) $$q_{ij}=\frac{{\left(1+{\left\|y_{i}-y_{j}\right\|}^{2}\right)}^{-1}}{\sum_{k\neq i}{\left(1+{\left\|y_{i}-y_{j}\right\|}^{2}\right)}^{-1}}$$

T-SNE optimizes the similarity between high and low dimensional spaces by minimizing Kullback–Leibler divergence (KL divergence). It uses gradient descent to adjust the position of points in low-dimensional space, which ensures that the similarity existing in high-dimensional space is preserved as much as possible.

(13) $$KL(P||Q)=\sum_{i}\sum_{j}p_{ij}log\frac{p_{ij}}{q_{ij}}$$

In evaluating the performance of the prediction model, the prediction results obtained from the model trained on the original data were compared with those from the model trained on the augmented data to demonstrate changes in model performance. The Root Mean Square Error (RMSE), Mean Absolute Error (MAE), and the coefficient of determination (R^2^ score) were used as metrics to assess the accuracy of the prediction models. RMSE reflects the magnitude of the deviation, MAE indicates the average level of error, and the R^2^ score shows the correlation between the predicted values and the actual values. The calculation methods for these evaluation metrics are defined as follows:

(14) $$RMSE=\sqrt{\frac{1}{n}\sum_{i=1}^{n}{\left(y_{i}-\hat{y_{i}}\right)}^{2}}$$

(15) $$MAE=\frac{1}{n}\sum_{i=1}^{n}\left|y_{i}-\hat{y_{i}}\right|$$

(16) $$R^{2}=1-\frac{\sum_{i=1}^{n}{\left(y_{i}-\hat{y_{i}}\right)}^{2}}{\sum_{i=1}^{n}{\left(y_{i}-\bar{y}\right)}^{2}}$$

where $y_{i}$ is the actual detected value, $\hat{y_{i}}$ is the predicted value of the model, and n is the number of samples. The smaller the values of RMSE and MAPE, the better the prediction performance of the representative model, and a larger value of R^2^ score indicates a stronger explanatory capability of the model.

### 3.4. Hyperparameters and Benchmarks

The deep learning model used for data augmentation consists of four components: embedding function, recovery function, generator, and discriminator. The generator and discriminator modules are implemented using a 3-layer GRU structure. The recovery module is achieved by combining RNN with a multi-head attention mechanism layer, and the embedding module is implemented using a 3-layer LSTM structure. The number of hidden units in the RNN layer is 64. The number of heads in the multi-head attention mechanism is 4. The loss function of the supervisor, embedding function, and recovery function is the mean square error, and the loss function of the generator and discriminator is binary cross-entropy. The model’s learning rate is set to 0.00003, with a batch size of 256 and a total of 800 iterations.

The model used for prediction consists of four components: the CNN layer, GRU layer, attention layer, and fully connected layer. In the model, the size of the time window is also set to 30 time steps. The weight calculation function of the CNN layer is LeakyReLU, the number of convolution layers is 1, and the number of hidden channels is set to 128. The number of GRU layers is 2, and Q, K, and V of the attention mechanism layer are all outputs of GRU layer. The learning rate is set to 0.0005, and the model is trained for 500 iterations.

### 3.5. Data Augmentation Result Analysis

**Figure 11:**
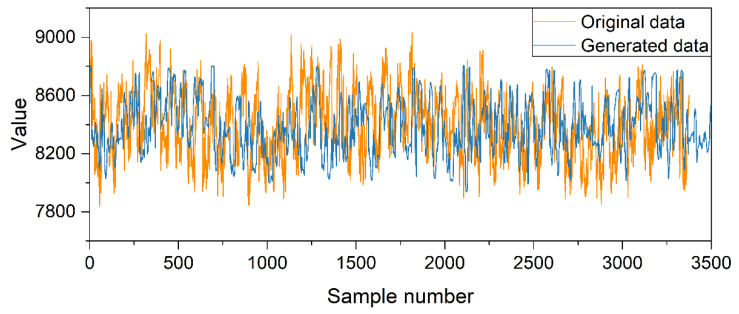
The original data and generated data.

After applying the improved TimeGAN model proposed in this paper for data augmentation, the data output by the model was denormalized to obtain the generated dataset. In terms of the overall trends of the time series, the situation of the fitted data compared to the real data is shown in Figure 11, where the yellow line represents the real data and the blue line represents the generated data. The relevant statistical feature parameters for both datasets are shown in Table 1. It can be observed that the statistical feature values of both datasets are quite similar. For the mean, the difference is 0.06% of the real data’s statistical parameter. For the standard deviation, the difference is 9.40%, and for the quartiles, the differences are 0.21%, 0.11%, and 0.35%, respectively. Additionally, the skewness and kurtosis values for the two datasets are also quite similar. This indicates that the generated data have a distribution trend similar to that of the original data in terms of overall statistical characteristics.

**Figure 12:**
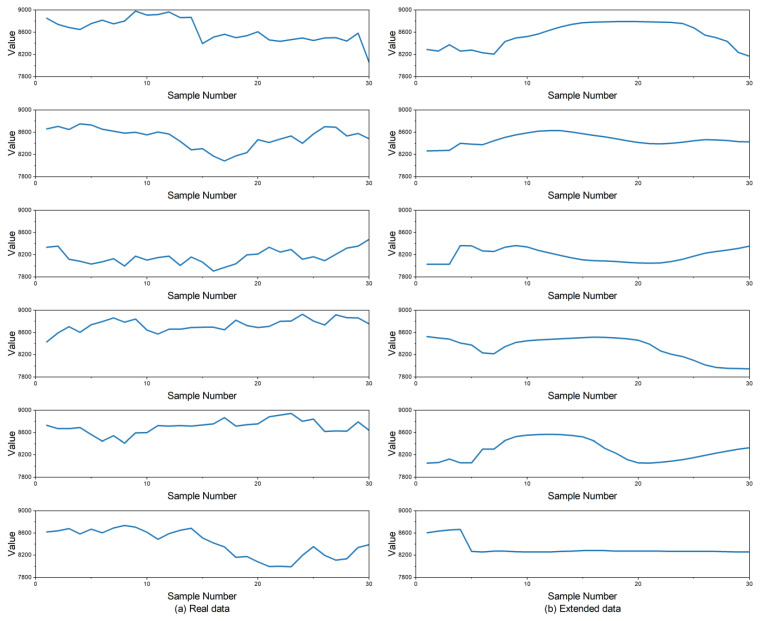
The data segments of the original data and generated data.

After segmenting the data into windows of size 30, 12 data fragments were intercepted in the original data and the generated data, respectively, which are shown in Figure 12. It can be observed that the overall fluctuation trends and data ranges of the original data and the generated data are similar; however, the generated data are smoother than the original data. This smoothness occurs because, during the data augmentation process, the generated data points align more closely with an “ideal” distribution instead of the true distribution of the original data. Even after data preprocessing, the original data may still have noise, outliers, or other irregularities. In the generated data, the continuous learning process of the neurons reduces the impact of these irregularities, and this results in a smoother fluctuation trend. During training, the deep learning model adjusts its weights with the back-propagation algorithm to minimize the loss function. Throughout training, the model often ignores minor fluctuations in the data; instead, it focuses on capturing the main trends. The data augmentation model generates samples that align with the overall trend in the original data rather than retaining all of the details from it.

**Figure 13:**
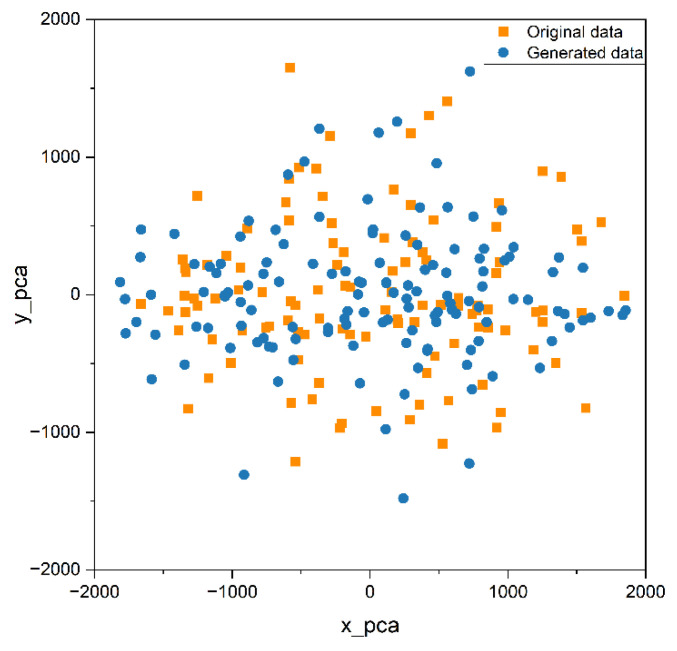
PCA visualization on the original data and generated data.

**Figure 14:**
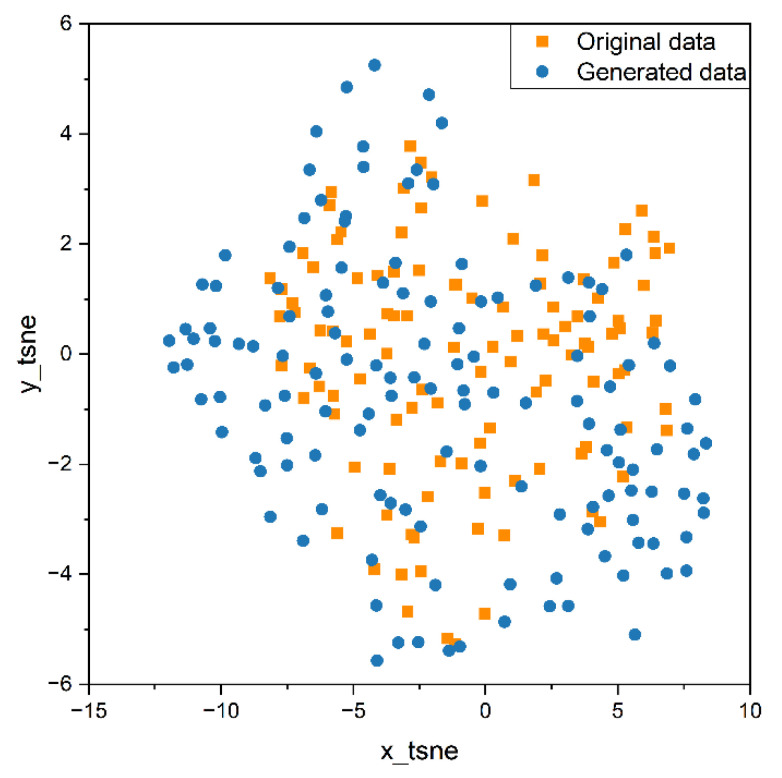
t-SNE visualization on the original data and generated data.

PCA and t-SNE were used to reduce the dimensions of the original and generated data segments for visual analysis and feature comparison. The results are shown in the scatter plots in Figure 13 and Figure 14. In these plots, yellow points represent the real data, while blue points represent the generated data. By looking at the distribution of points in the scatter plots, we can see that points from both datasets cluster in similar areas and have comparable distribution ranges, and there is no clear dividing line between them. Therefore, we can conclude that they have similar characteristics.

### 3.6. Prediction Model Result Analysis

**Figure 15:**
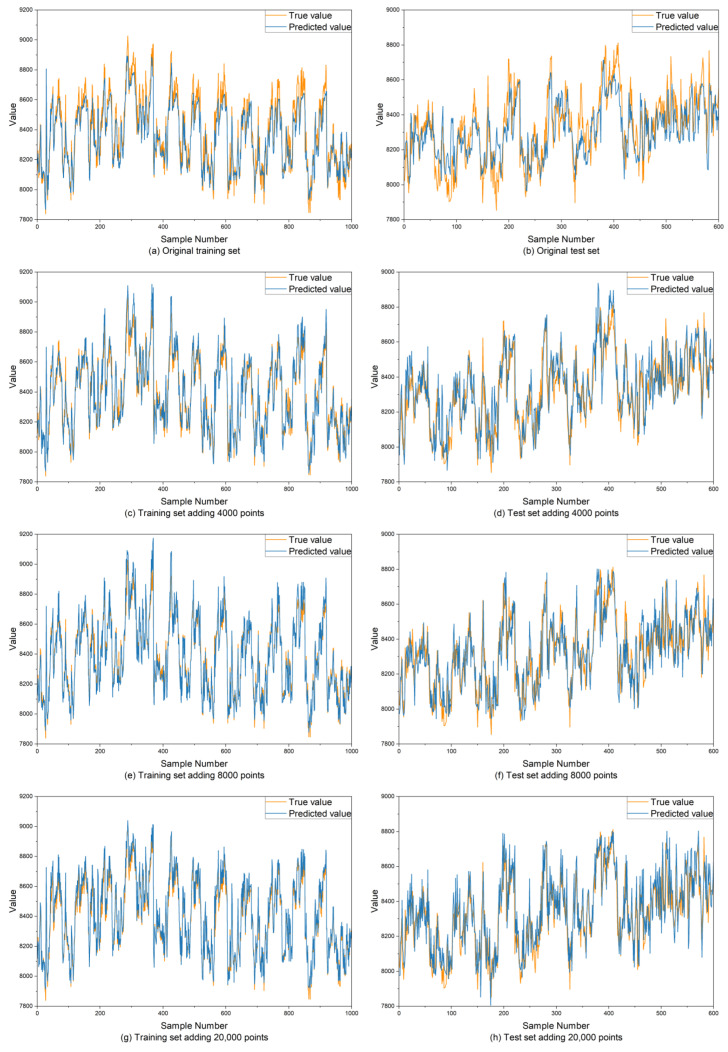
Results of prediction models on the training set and test set after using different amounts of generated data to expand the dataset.

The existing real dataset consisted of 3374 normal samples, with 80% of the data allocated to the training set and 20% reserved for the test set. After augmenting the training dataset with varying amounts of generated data, the performance of the prediction model was assessed by analyzing changes in the model’s prediction results. The prediction results of the model with different amounts of generated data are shown in Figure 15, where the yellow line represents the real data, and the blue line represents the prediction data. The comparative statistics are shown in Table 2.

As the amount of data in the training set increases, the RMSE and MAE of the prediction model on the training set continuously decrease, while the R^2^ value increases, demonstrating an improvement in the model’s fitting capability. However, in the test set, when the size of the generated data exceeds 8000, the judgment error of the prediction model tends to increase gradually. When the generated data size is increased from 8000 to 20,000, the improvement in the model’s prediction performance on the training set is not as pronounced as before, with RMSE and MAE decreasing by 19.78% and 6.81%, respectively, while R^2^ only increases 0.86%. On the test set, when the generated data size is 8000, the prediction error of the model is the smallest, and the fitting degree is the closest. However, when the size of the generated data is increased from 8000 to 20,000, RMSE and MAE increase by 12.70% and 11.61%, respectively, while R^2^ decreases by 0.64%. Therefore, it can be seen that when the generated data size is 8000, the expanded training set allows for the best comprehensive performance of the prediction model on the training set and the test set.

When the amount of generated data is limited, the lack of training samples is a key factor affecting model performance. Increasing the amount of generated data can improve the model’s prediction accuracy. However, when the amount of generated data exceeds three times that of the original data, most features learned by the model come from the generated data, which leads to a decline in performance on the test set. Therefore, after completing data augmentation, increasing the amount of training data indefinitely is not necessarily beneficial. Instead, the optimal amount of data augmentation should be determined based on the actual conditions of the model and the dataset.

**Figure 16:**
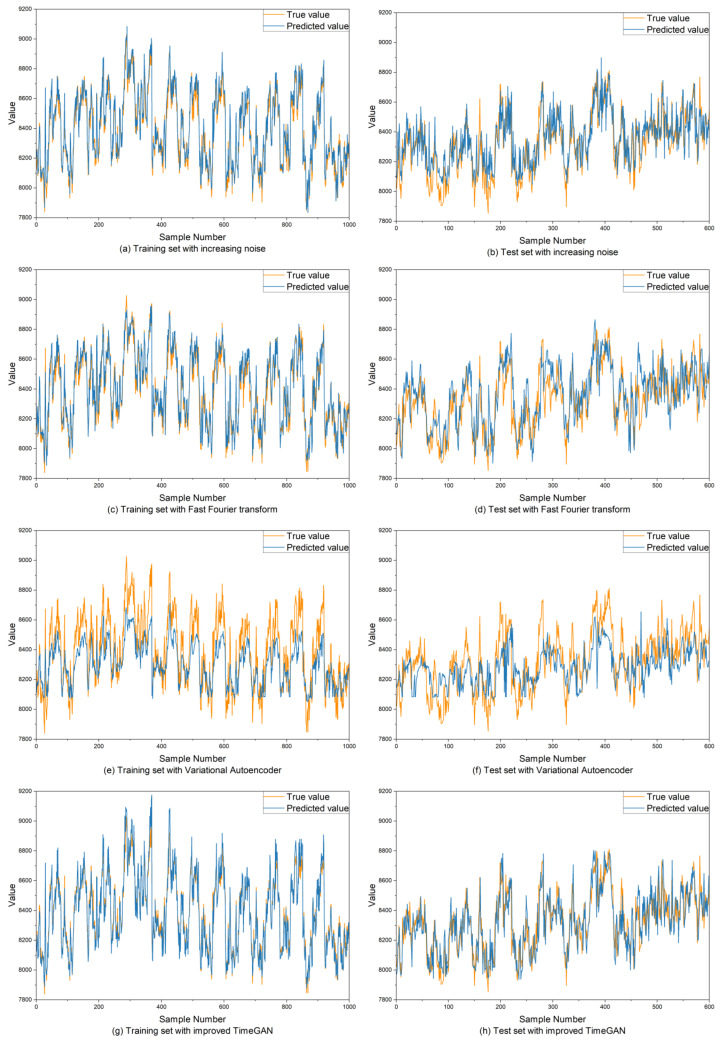
Results of prediction models on the training set and test set after using different data augmentation methods to expand the dataset.

In order to verify the data augmentation effect of the improved TimeGAN model proposed in this paper, two baseline algorithms for data augmentation were used for comparison. First, we added random noise to the original data to obtain generated data. Three different random number seeds were used to generate normal distribution noise, and then they were added to the original data respectively to obtain the generated data. At the same time, we transformed the original data into frequency domain components by fast Fourier transform [36], and then added random noise before inverse fast Fourier transform to generate new time series data. We also used a VAE model [15] for data augmentation to compare the performance of our proposed model. The above three data augmentation methods were used to expand 8000 pieces of data respectively, which were used to expand the training set of the prediction model. The model prediction results of different methods are shown in Figure 16, where the yellow line represents the real data and the blue line represents the predicted data. The comparative statistics are shown in Table 3. It can be seen that the RMSE and MAE of the improved TimeGAN model are the smallest and R^2^ is the largest in both the training set and the test set, which proves that it enables the smallest error of the prediction model and the closest fitting degree.

## 4. Conclusions

Predicting the energy consumption data of manufacturing process equipment is of significant importance for energy management and production control. Prediction algorithms based on deep learning models can accurately assess the trends in energy consumption data; however, they have high requirements for the quantity and quality of training data. To address the issue of limited energy consumption data collected from actual production lines, the improved TimeGAN model is employed for data augmentation of manufacturing equipment energy consumption data. This paper analyzes the similarity of features between the generated data and the original data and verifies the changes in the performance of the prediction model after incorporating the generated data into the dataset.

(1) The structure of the TimeGAN model is improved by incorporating a multi-head self-attention mechanism layer into the recovery module. As a result, the errors in the mean and quartiles between the generated data and the original data are maintained within 0.5%, while the variance error is kept within 10%. Additionally, the PCA and t-SNE dimensionality reduction analyses indicated that the trends in the data segments of the original data and the generated data are similar.
(2) After augmenting the training set size of the energy consumption time series prediction model with the generated data, the performance of the prediction model is significantly improved. Compared to the original model, the RMSE and MAE on the training set decrease by over 60%, while on the test set, they decrease by more than 9%. Additionally, the R^2^ score is also improved. The model trained after data augmentation better meets the requirements of model accuracy in the actual production process.
(3) This article validates that, when augmenting the training set of deep learning models using data augmentation, the quantity of generated data does not necessarily need to be maximized; rather, it should be analyzed based on the specific conditions of the dataset and the training model. In the context of the energy consumption time series in this paper, the prediction model performs optimally when the amount of generated data is set to 8000.

Testing the generalization ability of the data augmentation model on different datasets can further verify the practicability and reliability of the model. The influence of the size of the extended data on the performance of the prediction model is only tested on the current energy consumption dataset, and more experiments are needed to verify this conclusion. Grid search or Bayesian optimization can be used to find the best super parameter combination to improve the performance of the data augmentation model.

## Figures and Tables

**Table 1:** The comparison of statistical parameters.

Index	Original Data	Generated Data
Average	8401.65	8396.60
Standard deviation	225.00	203.86
1st quartile	8231.02	8248.70
2nd quartile	8401.27	8392.21
3rd quartile	8567.21	8537.59
Skewness	0.0652	0.0790
Kurtosis	0.5908	0.7221

**Table 2:** The prediction results of different prediction models.

Generated Data Size	Training Set			Test Set		
	RMSE	MAE	R^2^	RMSE	MAE	R^2^
0	88.6316	70.0475	0.8465	131.3622	101.3626	0.5653
4000	58.0971	40.9832	0.9238	123.5372	96.989	0.6296
8000	35.0361	24.4527	0.9728	118.445	91.8783	0.6327
12,000	46.8471	38.7948	0.9506	128.5719	100.243	0.5757
16,000	33.1651	22.4858	0.9745	129.3287	100.1522	0.6132
20,000	28.1058	22.7874	0.9814	133.4881	102.5445	0.6263

**Table 3:** The prediction results of different data augmentation methods.

Data Augmentation Methods	Training Set			Test Set		
	RMSE	MAE	R^2^	RMSE	MAE	R^2^
Increasing noise	62.8416	48.9257	0.9348	121.9678	95.273	0.6105
Fast Fourier transform	54.1052	41.3113	0.9547	139.6714	108.2355	0.4892
Variational Autoencoder	146.1772	98.5957	0.8288	155.2344	124.1232	0.3691
Improved TimeGAN	35.0361	24.4527	0.9728	118.445	91.8783	0.6327

## Data Availability

Data are contained within the article.
